# The Temporal and Spatial Distributions and Influencing Factors of Transboundary Pollution in China

**DOI:** 10.3390/ijerph19084643

**Published:** 2022-04-12

**Authors:** Zhonghao Zhang, Tiantian Nie, Yingtao Wu, Jiahui Ling, Danhuang Huang

**Affiliations:** 1 School of Environmental and Geographical Sciences, Shanghai Normal University, Shanghai 200234, China; zzh87@shnu.edu.cn (Z.Z.); 1000496515@smail.shnu.edu.cn (T.N.); 2 Shanghai Key Lab for Urban Ecological Processes and Eco-Restoration, East China Normal University, Shanghai 200241, China; 3 Wetlands Ecosystem Observation and Research Field Station, Shanghai Normal University, Shanghai 200234, China; 4 School of Finance and Business, Shanghai Normal University, Shanghai 200234, China; lingjiahui1997@163.com (J.L.); danhuang_huang94@163.com (D.H.); 5 Global Innovation Capital Research Institute, Shanghai Normal University, Shanghai 200234, China

**Keywords:** environmental pollution, boundary effect, geographical distribution, China

## Abstract

Transboundary pollution between neighboring regions seriously affects the efficiency of overall environmental governance; however, there are few studies focused on how to estimate the degree of transboundary pollution between different regions. With China as a case study, this article developed a new measurement to estimate the degree of transboundary pollution among regions, and comprehensively investigated the influencing factors of transboundary pollution in China between 2000 and 2013. The results indicate that transboundary pollution effects exist in China. In ascending order, the regions most affected overall by transboundary pollution from polluting enterprises were as follows: eastern region < central region < western region. The reduction in transboundary pollution effects was most prominent for severely polluting enterprises in the eastern and western regions and lightly polluting enterprises in the central region. An analysis of the influencing factors reveals that the regional environmental regulation intensity has a negative feedback effect on the transboundary pollution effects. These findings indicate that polluting enterprises in regions with a low environmental regulation intensity are more inclined to operate in border areas to obtain both the environmental benefits associated with the low local environmental regulation intensity and the market economy benefits associated with neighboring urban regions, thereby aggravating environmental pollution in border areas.

## 1. Introduction

With rapid economic development, environmental pollution has become increasingly intense and widespread in developing countries [1,2]. Although the Chinese government has implemented extremely strict environmental governance policies, environmental problems remain serious, and environmental governance is increasingly difficult [3]. In addition to the inertia sources of pollution, the environmental policy intensity, governmental enforcement, and other factors associated with territorial economic growth encourage local governments to take advantage of the spillover effects and externalities of pollution by locating polluting enterprises along administrative boundaries, where some of the pollution will affect neighboring regions [4,5,6,7], a phenomenon referred to as the transboundary pollution effect. This transfer of pollution has not effectively improved environmental quality [8] and is not conducive to the further implementation and improvement of environmental governance policies [9].

Transboundary pollution between countries has been studied for many years, and most studies have been based on the frameworks of general equilibrium analysis or game theory [10,11,12,13]. Relevant studies have shown that transboundary pollution between countries is mainly achieved by increasing the height of chimneys to transfer some of the pollution to other countries to reduce domestic pollution, or by positioning polluting enterprises in border regions [14]. Moreover, when policy regulation is relatively weak, governments of states adjacent to a border are more motivated to promote border pollution [15]; i.e., more polluting enterprises are located in counties bordering neighboring countries to gain the benefits of domestic economic activities while forcing neighboring countries to bear the resultant environmental costs [16,17]. Additionally, the problem of boundary pollution between neighboring regions (province/city or state/county) within a country has also received an increasing amount of attention [18,19]. Relevant studies have shown that significant transboundary pollution effects are generally present between neighboring regions in a country and are closely related to the degree of difference in the environmental regulation intensity between regions [20] and coordination costs [12]. In other words, the greater the difference in the environmental regulation intensity between regions and the higher the coordination costs, the greater the transboundary pollution effects. However, when the coordination costs are reduced to a certain level, the transboundary pollution effects become insignificant [21,22].

Based on China’s political centralization and regional economic decentralization, the environmental pollution problem in border areas between neighboring provinces/cities may be more serious. Duvivier and Xiong [5] considered Hebei Province as an example and found that polluting enterprises were more likely to be established in border counties between neighboring provinces/cities than in inland areas. Kahn et al. [23] found that the water pollution in border areas in China is more serious than that in the inland areas of provinces/cities based on a difference-in-differences model. Cai et al. [24] and Zhang et al. [25] further analyzed the formation mechanism of the transboundary pollution effects of rivers in China and determined that the effect is mainly due to the strict water quality control and reform policies that provinces directly manage in the counties under their jurisdiction in China.

Although extensive and in-depth studies have been conducted on transboundary pollution, there are still many issues that need to be addressed and improved. For example, from the perspective of research methods, most studies have verified the existence of transboundary pollution through a general equilibrium theory model, a difference-in-differences model, and other econometric models [10,23,26]. There has been a general lack of consideration of the degree of transboundary pollution. From the perspective of the spatial scale, most studies have been conducted within the regions of a country (such as China) [5], and comparing the differences in the transboundary pollution effects between regions is difficult. From the perspective of the temporal scale, the number of studies on the temporal trends of transboundary pollution effects is limited. From the perspective of influencing factors, although it has been proven that the implementation of environmental regulations can lead to the “pollution paradise” effect in border areas, the difference in the implementation and environmental regulation intensity between regions may merely change the spatial distribution of pollution [27,28,29,30,31], and its effect on transboundary pollution remains unclear, which is not conducive to proposing policy recommendations targeting reductions in transboundary pollution effects.

Therefore, China, a developing country with a severe pollution situation, was used as an example in this study to investigate the average degree, spatial distribution, and temporal trend of transboundary pollution effects in various regions of China, and to explore the main influencing factors. The contribution of this study mainly lies in three aspects (1) Research method and spatial scale: This study combined a geographic information system (GIS) method and the China Industrial Enterprise Database to obtain the spatial distribution of industrial enterprises above the designated size (including polluting enterprises) and the distribution of provincial and city borders in China to construct measurement indices for evaluating the degree of transboundary pollution and to investigate the spatial distribution and dynamic changes in the degree of transboundary pollution in China. (2) Classification and comparison of the degree of transboundary pollution: The pollution efficiency of different types of polluting enterprises was distinguished. All polluting enterprises were classified into three different pollution severities (severe, moderate, and light) based on their degrees of pollution and into three spatial types (eastern, central, and western), based on their locations, to analyze the migration pattern of enterprises with different pollution types and the changes in the transboundary pollution effects in different regions of China. (3) Analysis of the influencing factors of transboundary pollution effects: Based on the magnitude of the transboundary pollution effects in various regions of China, the main influencing factors, including environmental regulations, regional economic conditions, and traffic conditions, were explored based on regional integration. Targeted decision-making opinions were proposed based on regional integration and the joint prevention and control of environmental pollution. This approach is conducive to the implementation of environmental governance policies in different regions that promote simultaneous improvements in the overall environmental quality and sustainable and stable economic development. In addition, this approach can be used to further mitigate transboundary pollution and improve the overall quality of regional environments in the future. The research content and methods used in this study have general relevance and can be easily extended to research on transboundary pollution problems in other countries or regions, thus providing a reference for other studies in this field.

## 2. Materials and Methods

### 2.1. Measurement Method for the Degree of Transboundary Pollution and an Empirical Model of Its Influencing Factors

#### 2.1.1. Measurement Method for the Degree of Transboundary Pollution

To prevent the error caused by the use of absolute values, the existence and degree of transboundary pollution were evaluated by measuring the relative proportion of the scale of polluting enterprises in border areas and non-border areas in the same province.

First, based on GIS spatial software, the geographic locations of all the industrial enterprises above a designated size in China were obtained, and the number of enterprises in each region was determined. Next, the proportion of polluting enterprises in border areas of each province ($border_{jt}/border_{ft}$), and the proportion of polluting enterprises in non-border areas of the corresponding province ($norbor_{jt}/norbor_{ft}$) were calculated. Finally, the transboundary pollution effects in a region ($effect_{jt}$) are expressed as the ratio of the proportion of polluting enterprises in border areas to the proportion of polluting enterprises in non-border areas. The degree of the transboundary pollution effect is calculated using Equation (1):

(1) $$effect_{jt}=\frac{border_{jt}/border_{ft}}{norbor_{jt}/norbor_{ft}}$$

where $border_{jt}$ refers to the sum of the total assets of the enterprises of pollution type j in border counties in year t, and $border_{jt}$ refers to the sum of the total assets of all the enterprises (including polluting and nonpolluting enterprises) in the border counties in year t.

(1) Transboundary pollution effect:

If $effect_{jt}$ is greater than 1, the scale of the polluting enterprises in the border area of the province is greater than that in the non-boundary area. Therefore, there are transboundary pollution effects in the province, which may be due to the transfer of polluting enterprises in the non-boundary area to the boundary area, or the new scale of the polluting enterprises in the boundary area is greater than that in the non-boundary area. Comparing the value of $effect_{jt}$ between different provinces can determine which province has greater transboundary effects. Conversely, if $effect_{jt}$ is less than 1, there is no transboundary pollution effects.

(2) Degree of transboundary pollution:

The value of $effect_{jt}$ is directly proportional to the degree of transboundary pollution. The larger $effect_{jt}$ is, the greater the degree of transboundary pollution is, and the smaller $effect_{jt}$ is, the lesser the degree of transboundary pollution is.

#### 2.1.2. Empirical Model of Factors Influencing Transboundary Pollution

In this study, the calculated average degree of transboundary pollution in each province is used as the explanatory variable, the environmental regulation intensity is selected as the core explanatory variable, and the relevant control variables that affect the regional average transboundary pollution effects are selected to build an econometric model. The regression model is described as follows:

(2) $$\ln effect_{it}=\beta_{1}\ln ERS_{it}+\beta_{2}\ln eco_{it}+\beta_{3}\ln way_{it}+\beta_{4}\ln edu_{it}+\beta_{5}\ln pop_{it}+\beta_{6}\ln RD_{it}+\varepsilon_{it}$$

where $effect_{it}$ is the average degree of transboundary pollution in province i in t years; $ERS_{it}$, $eco_{it}$, $way_{it}$, $edu_{it}$, $pop_{it}$, and $RD_{it}$ represent the regional environmental regulation intensity, the regional economic development level, the regional transportation convenience, the regional education level, the regional market size, and the regional R&D and innovation level of province i in year t, respectively; $\beta_{1}\sim \beta_{6}$ are the respective regression coefficients; $\varepsilon_{it}$ is a random error term. Considering that the data may have heteroscedasticity, all variables were corrected by logarithmic processing.

### 2.2. Data Description and Index Selection

#### 2.2.1. Data Sources

Based on the data availability for industrial enterprises in the China Industrial Enterprise Database, the years 2000 and 2013 were selected.

The 2000–2013 period was a critical period in China’s environmental regulation and economic development. Environmental protection was first mentioned in China’s 10th Five-Year Plan in 2001. Therefore, the year 2000, before the announcement of the policy, was selected as the initial time node of this study. The year 2012 was the last year of the golden decade of economic growth in China during the Hu-Wen era, and 2013 was selected as the final time node of this study to explore the shifting trend of environmentally polluting industries in the context of rapid economic growth.

Although there is competition for jurisdiction among local governments at all levels, the competition between provincial governments is particularly fierce in terms of economic growth and environmental quality [9]. Based on a study by Xin and He [32], provincial border areas were classified into eastern, central, and western regions to analyze the changes in the transboundary pollution effects in different regions. The regional division of China is shown in Table 1.

The longitude and latitude of all enterprises (including polluting and nonpolluting enterprises) in 2000 and 2013 were obtained through the Baidu map API interface. Enterprise codes, enterprise names, industry codes, administrative division codes and total assets indices were obtained from the China Industrial Enterprise Database. Then, the total assets of the enterprises with different pollution types in each county were calculated by ArcGIS 10.7. By comparing the proportion of polluting enterprises between counties (border layer) on the provincial boundary and counties (non-border layer) within the province, the administrative boundary effect of Chinese enterprise pollution was analyzed.

**Figure 1:**
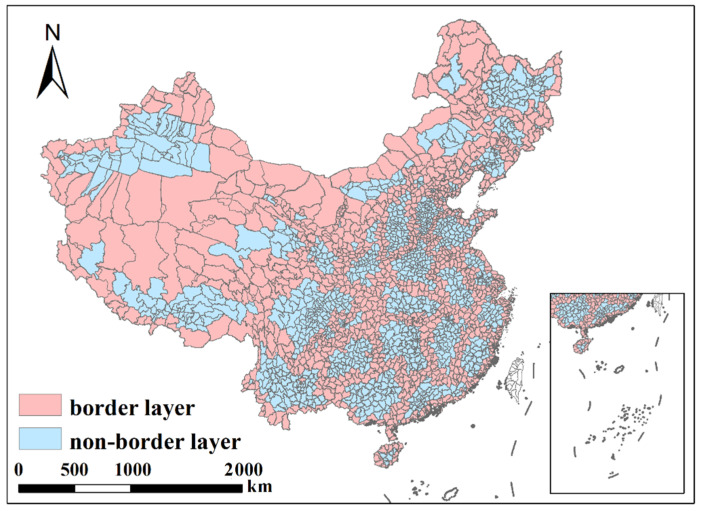
Division of polluted areas: border layer and non-border layer.

As shown in Figure 1, the border layer contains 1135 county-level cities, shown in pink; the non-border layer contains 1246 county-level cities, shown in azure (excluding Hong Kong, Macao, and Taiwan).

#### 2.2.2. Definition of Polluting Industries and Data Description

Based on the classification method for polluting industries used by Wang and Xia [33], this study uses industrial waste gas emissions, sulfur dioxide emissions, industrial wastewater emissions and the total industrial output value to calculate the pollutant emission intensity coefficients for different industries, and classify the industries into severely, moderately, and lightly polluting industries. The classification results are shown in Table 2.

#### 2.2.3. Analysis of the Factors Influencing the Transboundary Pollution Effects

Core explanatory variable: Environmental regulation intensity (lnERS): The regional environmental regulation intensity is an important factor that affects the site selection of polluting enterprises. Facing the constraints of a high environmental regulation intensity, polluting enterprises may relocate their production and operation sites to regions with a lower environmental regulation intensity. Under the strict constraints of environmental governance, this variable is expected to have a significantly negative impact on the transboundary pollution effects.

(1) Control variables: Based on past literature [21,22], the possible profit of an enterprise is the traditional determinant of site selection.
(2) Regional economic development level (lneco): In areas with low levels of economic development, local governments tend to neglect to control polluting enterprises to accelerate local economic development. The low pollution control cost intensifies the tendency of enterprises to select production sites at border areas. This variable is expected to have a negative impact on the transboundary pollution effect.
(3) Regional transportation convenience (lnway): The more convenient the transportation in a region is, the higher the tendency of enterprises to select production sites in the region to reduce production costs. This variable is expected to have a positive impact on the transboundary pollution effect.
(4) Education level (lnedu): Regions with higher levels of education have more high-quality labor and higher levels of productivity. This variable is expected to have a negative impact on the transboundary pollution effect.
  Market size (lnpop): A large labor market in a region implies a low cost for human resources and indicates the presence of more production resources in the region, which encourages polluting enterprises to choose sites in this area. This variable is expected to have a positive impact on the transboundary pollution effect.
(5) Market size (lnpop): A large labor market in a region implies a low cost for human resources and indicates the presence of more production resources in the region, which encourages polluting enterprises to choose sites in this area. This variable is expected to have a positive impact on the transboundary pollution effect.
(6) R&D and innovation level (lnRD): The scientific and technological progress due to R&D and innovation is conducive to bringing more advanced and environmentally friendly production technologies to a region and promoting improvements in the regional environmental quality. This variable is expected to have a negative impact on the transboundary pollution effect. The calculation methods for the above indices are provided in Table 3.

## 3. Analysis of the Temporal and Spatial Trends of Transboundary Pollution Effects in China

### 3.1. The Temporal Trend of the Overall Transboundary Pollution Effects in China

The transboundary pollution effects of enterprises in 2000 and 2013 and their dynamic changes were calculated and compared. First, the calculated data for each province/city were standardized to the national level. Then, the transboundary pollution situations of enterprises of different pollution types were calculated based on the classification in Table 2. The results are shown in Table 4.

The results in Table 4 indicate that the degree of the overall transboundary pollution effects of polluting enterprises in 2000 and 2013 was greater than 1, suggesting overall transboundary pollution effects. The transboundary pollution impact of seriously polluting enterprises decreased significantly from 2000 to 2013, with a decline rate of 50.76%, which may be related to the significant reduction in the total number of seriously polluting enterprises caused by China’s industrial transformation and upgrades. In 2000 and 2013, the transboundary pollution impact of moderately polluting enterprises was greater than 12,000, and the transboundary pollution impact increased by 13.41% in 2013. In 2000 and 2013, the transboundary pollution impact of lightly polluting enterprises was greater than 1. From 2000 to 2013, the transboundary pollution impact decreased only slightly. This finding shows that although the transboundary pollution of China’s seriously polluting enterprises has decreased, there is still regional cross-border pollution in China. Transboundary pollution is mainly caused by moderately and lightly polluting enterprises rather than severely polluting enterprises.

In summary, there were transboundary pollution effects in China during the 2000–2013 period. In other words, the scale of polluting enterprises on the provincial boundary was larger than that in non-boundary areas. This result shows that China’s environmental policies can involuntarily lead to boundary effects, pollution has negative externalities, and that environmental regulation is discontinuous in the same administrative jurisdiction [16], resulting in a “free ride” effect. Polluting enterprises are affected by policies. To reduce their own environmental treatment costs, these enterprises are more willing to select sites in border areas with less strict environmental regulations [5], especially for moderately polluting enterprises.

### 3.2. The Spatial Trend of Transboundary Pollution Effects in China

**Figure 2:**
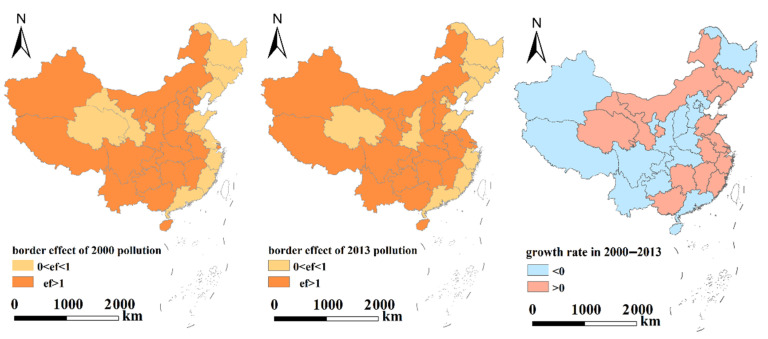
Transboundary pollution effects at the provincial level (all types of polluting enterprises).

**Figure 3:**
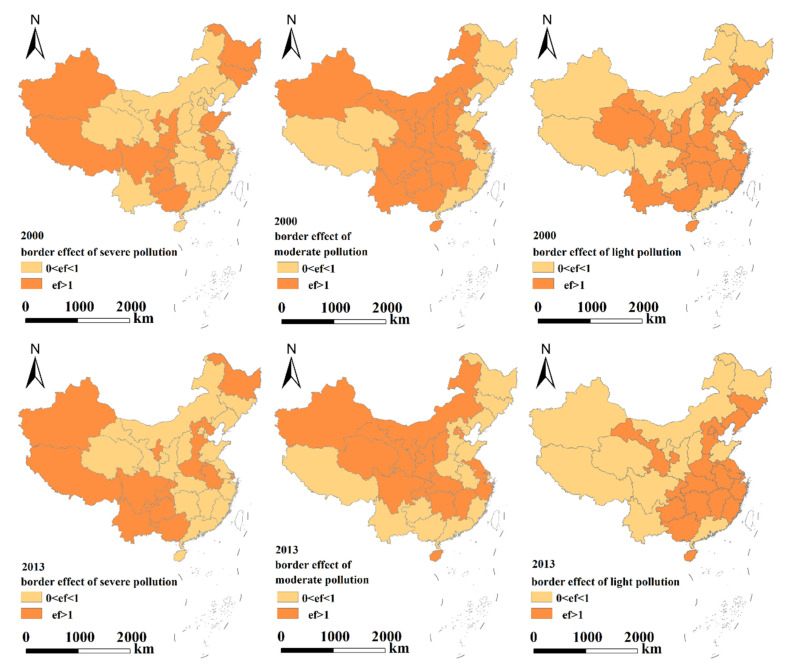
Transboundary pollution effects at the provincial level (three types of polluting enterprises).

**Figure 4:**
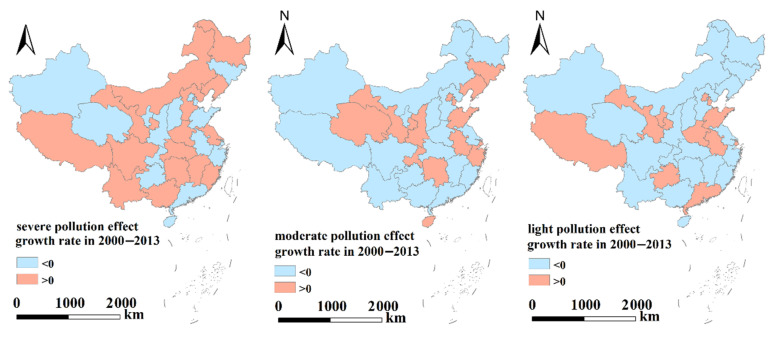
Growth rate of transboundary pollution effects at the provincial level in 2000–2013 (three types of polluting enterprises).

Considering the regional differences in economic development and environmental policies in eastern, central and western China, the boundary effects of enterprises with different pollution types were analyzed. The transboundary pollution effects were further refined to the provincial/municipal level, and the transboundary pollution in each province/city was analyzed (Table 5 and Figure 2, Figure 3 and Figure 4).

Table 5 and Figure 2, Figure 3 and Figure 4 indicate the following.

(1) Eastern region: From the perspective of various polluting enterprises, the impact of cross-border pollution gradually weakened, severely polluting enterprises had no cross-border pollution impact in 2013, and the cross-border pollution effect of moderately polluting enterprises showed positive growth. Although the cross-border pollution impact of lightly polluting enterprises has weakened, it is still serious.
  The transboundary pollution effects of severely polluting enterprises in Beijing, Tianjin, Shandong, Zhejiang, Guangdong and Hainan, moderately polluting enterprises in Hebei, Jiangsu, Fujian, Guangdong and Jiangxi, and lightly polluting enterprises in Liaoning, Hebei, Jiangsu, Zhejiang, Fujian, Jiangxi and Hainan gradually weakened, indicating that the choice of geographical locations for different polluting enterprises in these cities/provinces has been subject to certain restrictions and constraints.
(2) Central region: Although the transboundary pollution effects of all types of polluting enterprises, especially moderately and lightly polluting enterprises, gradually weakened, the overall transboundary pollution effects were still prominent. The transboundary pollution effects of severely polluting enterprises increased slightly. The transboundary pollution effects of severely polluting enterprises showed negative growth in Anhui and Shanxi and positive growth in all other provinces/cities, indicating that the severely polluting enterprises in most provinces/cities in the central region were still more inclined to choose sites in border areas. The choice of geographical location for polluting enterprises was not subject to restrictions and controls.
(3) Western region: Although the transboundary pollution effects of all severely and moderately polluting enterprises decreased, the transboundary pollution problem was still prominent. This finding indicates that although the size of the polluting enterprises in the western region decreased, transboundary pollution in the western region is still an issue due to the influences of different natural environments and policies on enterprises. The transboundary pollution effects of the lightly polluting enterprises in Guizhou, Ningxia, Gansu, and Tibet increased slightly. Among all the polluting enterprises, the transboundary pollution effects of the severely polluting enterprises decreased to the greatest extent.

Regardless of the type of polluting enterprises, the order of the cross-border pollution impact intensity was Western > Central > Eastern, the order of the cross-border pollution impact intensity of seriously polluting enterprises was Western > Central > Eastern, the order of moderately polluting enterprises was Eastern > Western > Central, and the order of lightly polluting enterprises was Eastern > Western > Central. These results show that severely polluting enterprises have tended to choose locations in the western region, while moderately and lightly polluting enterprises have tended to choose locations in the eastern region. The increase in environmental supervision by local governments has indirectly promoted the location and production of polluting enterprises in border areas. During the study period, the cross-border pollution effects of moderately polluting enterprises in the eastern region, seriously polluting enterprises in the central region and lightly polluting enterprises in the western region showed positive growth.

## 4. Empirical Analysis of the Factors Influencing Transboundary Pollution Effects in China

In this study, panel data were used to analyze the impact of environmental regulations and other variables on regional transboundary pollution effects. First, the fixed effects model and the random effects model were used for regression analysis. Second, to ensure the validity of the parameter estimation, the Hausman test was performed on the regression results. The results are shown in Table 6; the F values of the overall regression of the model are all less than the significance level of 0.01, indicating that the model is overall significant.

The results of the Hausman test, i.e., a *p* value of 0.1963, indicated that the random effects model was more appropriate than the fixed effects model for explaining the regression results. Therefore, the following analyses and explanations were based on the estimation results of the random effects model (2). The specific analyses were as follows:

(1) The environmental regulation intensity had a significantly negative impact on the regional average transboundary pollution effect, indicating that the lower the regional environmental regulation intensity was, the larger the transboundary pollution effect was. Generally, regions with a relatively low environmental regulation intensity have relatively low economic levels, with an imperfect market industry structure. Polluting enterprises in these regions were more inclined to select sites in border areas to obtain the environmental benefits associated with the low local environmental regulation intensity and the market economy benefits associated with neighboring urban areas, thereby increasing the degree of environmental pollution in these border areas. Second, in the face of high environmental regulation intensity constraints, polluting enterprises may move their production and operation sites to regions with a low environmental regulation intensity, effectively reducing the local transboundary pollution effect.
(2) The regional economic development level had a notably negative impact on the transboundary pollution effect. On the one hand, regions with a low economic development level have a low degree of marketization, and it is difficult to effectively allocate the demand and supply of resources in border areas, thereby aggravating pollution in these areas. On the other hand, areas with a low economic development level generally have weaker environmental governance. The negative externality of pollution is more likely to stimulate the free-riding behavior of polluting enterprises. To improve their own economic benefits, enterprises tend to emit pollution to other regions and thus choose sites in border areas [5].
(3) The regional transportation convenience had a remarkably positive impact on the transboundary pollution effect, indicating that the completeness of the infrastructure in a region is an important factor that affects site selection by traditional enterprises. Enterprises are more inclined to choose sites in border areas with more complete infrastructure, thereby exacerbating the pollution level in border areas. In fact, the level of transportation infrastructure in counties on provincial borders is even lower [34], and the investment decisions of provincial governments do not fully consider the production endowment of border areas and the externalities of infrastructure.
(4) The education level had a greatly negative impact on the transboundary pollution effect, indicating that the transboundary pollution effects are weaker in regions with more high-quality labor or higher levels of productivity, potentially because high-quality laborers with higher education levels are employed by tertiary industries that have relatively limited impacts on pollution. An increase in high-quality labor resources is conducive to reducing the transboundary pollution effect.
(5) The market size was positively correlated with the regional transboundary pollution effect, indicating that the larger the market size is and the higher the population density is in a region, the more resources are needed in the region, thus increasing the willingness of polluting enterprises to choose sites in the region and exacerbating transboundary pollution. In addition, the larger the market size in a border area is, the greater the economic benefits that polluting enterprises can obtain from the border area are, and the more severe the pollution in the border area is.
(6) The R&D and innovation level had an evidently negative impact on regional transboundary pollution effects, indicating that improvements in the regional R&D and innovation capacity can raise the regional scientific and technological innovation level, optimize the production of polluting enterprises, and introduce green technology, all of which are conducive to reducing pollutant emissions in border areas [35].

The impact of the above factors lays a foundation for the subsequent reduction in the degree of transboundary pollution and for more targeted policy recommendations.

## 5. Discussion

### 5.1. Exploring the Transboundary Pollution Effects: A Unified Method Focusing on the Degree Measurement

Attention to transboundary pollution has been shifting from transboundary pollution between countries to transboundary pollution between regions within countries. However, there is no unified method to measure transboundary pollution, and there is a lack of research on the degree of transboundary pollution [36]. In this study, a new measurement method for transboundary pollution effects that can easily clarify the presence and degree of transboundary pollution was developed. The transboundary pollution problem between provinces and cities in China was empirically measured by comparing the relative distribution densities of polluting enterprises in border and non-border counties based on a GIS analysis method and enterprise microdata.

Compared to the methods used in previous studies on transboundary pollution in China [5], the proposed method can be used to compare the degree and trend of transboundary pollution between different provinces/cities/regions. Polluting enterprises were classified based on different pollution degrees to obtain a more comprehensive understanding of the pattern and characteristics of transboundary pollution in China.

By comparing the degree of cross-border pollution in the eastern, central and western regions, the cross-border pollution differences in areas with great differences in economic development levels, environmental governance levels and geographical resources were evaluated [37]. For example, in the eastern region, as of 2013, severely polluting enterprises had no cross-border pollution impacts, but moderately and lightly polluting enterprises still had serious cross-border pollution impacts. To some extent, this finding reflects the problems associated with China’s industrial transformation and upgrading and the impact of local environmental regulations [38].

### 5.2. The Implications and Further Perspective of the Proposed Measurement Method

We hope to study the causes of cross-border pollution and gradually eliminate it. Therefore, it is necessary to conduct an empirical analysis on the main influencing factors of transboundary pollution. The acquisition of panel data on the degree of transboundary pollution enabled us to comprehensively study the impact of transboundary pollution. Compared with previous studies that only focused on a single influencing factor [21,25], our analysis is clearer and more comprehensive. For example, the difference in environmental regulation between neighboring regions is an important influencing factor. Polluting enterprises prefer to choose areas with a low environmental supervision intensity to obtain environmental benefits generated from that low environmental supervision intensity and the economic benefits generated through adjacent cities [38,39]. Therefore, this factor implicitly promoted the transfer of polluting enterprises to areas with a low environmental regulation intensity, thereby aggravating the environmental regulation intensity in border areas. The transportation convenience, economic level, and innovation level also had significant impacts on transboundary pollution. Therefore, the results of this study can provide a reference for formulating more targeted policies. For example, research has shown that the administrative boundary of pollution should be abolished to strengthen coordinated environmental governance to reduce pollution at its source [40]. Regional technological progress and talent introduction can promote the upgrading of the industrial structure and the gathering of high-quality talent, reduce the impact of cross-border pollution, and achieve the targeted management of environmental pollution in border areas.

## 6. Conclusions

From the perspective of the geographical distribution of pollution caused by industrial enterprises, this study investigated the temporal and spatial changes in transboundary pollution in China from 2000 to 2013, using GIS tools and an innovative measurement method for transboundary pollution effects, and analyzed the main influencing factors of transboundary pollution. This study provides a reference for understanding the degree and trend of transboundary pollution in various regions of China and for formulating rational control policies; it also provides a research method and a research perspective for studying transboundary pollution in other regions of the world. The main conclusions of this study are as follows.

(1) From the perspective of temporal trends, there were significant transboundary pollution effects in China from 2000 to 2013, but the overall trend of transboundary pollution was attenuated. Notably, severely polluting enterprises in some areas no longer generated any transboundary pollution effect, which is related to the Chinese government’s strengthening of environmental regulations and the increasing degree of cross-regional coordination.
(2) In terms of spatial distribution, the overall transboundary pollution effects of polluting enterprises in the central region were stronger than those in the western and eastern regions, and the reduction in the overall transboundary pollution effects was most prominent for severely polluting enterprises in the eastern and western regions and for moderately and lightly polluting enterprises in the central region. This is related to China’s regional industrial transfer and the construction of an ecological civilization. For example, due to the industrial upgrading in the eastern region, many heavily polluting enterprises in the eastern region have moved to the central and western regions, thus strengthening the ecological environment control in the western region.

The problem of transboundary pollution reduces the overall effect and efficiency of environmental pollution control and seriously affects regional social welfare. This situation is conducive to environmental pollution control and may lead to a series of social events, such as environmental disputes in border areas. Therefore, according to the influencing factors of cross-border pollution, the following suggestions are proposed:

(1) Establish a more flexible environmental decentralization system and a comprehensive central supervision mechanism; strengthen the coordination mechanism of environmental governance among local governments; break administrative boundaries; support the cooperation of local governments to control regional environmental pollution problems; promote the integration of human and material resources; and reduce pollutant emissions from the source.
(2) Improve the independence of environmental protection departments; prevent speculative behavior by local pollution control officials; and develop the concept of “economy + environment”.
(3) The introduction of high-quality talent in border areas should be accelerated, the per capita education level in border areas should be improved, and the overall environmental awareness of border residents should be strengthened. The transformation of polluting enterprises from passive pollution control to active pollution control should be promoted so that polluting enterprises can take the initiative to improve the environmental quality in border areas. Furthermore, the environmental supervision function of residents in border areas should be promoted to restrict the transboundary pollution tendencies of local governments and enterprises.

## Figures and Tables

**Table 1:** Regional division of China.

Region	Included Provinces and Cities
Eastern region	Liaoning, Beijing, Tianjin, Hebei, Shandong, Jiangsu, Shanghai, Zhejiang, Fujian, Guangdong, Hainan
Central region	Heilongjiang, Jilin, Shanxi, Henan, Anhui, Hubei, Jiangxi, Hunan
Western region	Inner Mongolia, Ningxia, Xinjiang, Shaanxi, Gansu, Qinghai, Tibet, Chongqing, Sichuan, Guizhou, Guangxi, Yunnan

**Table 2:** Classification of polluting industries.

Classification	Polluting Industries
Severely polluting industries (6)	Mining and Processing of Nonferrous Metal Ores, Mining and Processing of Ferrous Metal Ores, Production and Supply of Electric Power and Heat Power, Smelting and Pressing of Ferrous Metals, Manufacture of Paper and Paper Products, Mining of Other Ores
Moderately polluting industries (4)	Manufacture of Raw Chemical Materials and Chemical Products, Smelting and Pressing of Nonferrous Metals, Mining and Washing of Coal, Manufacture of Nonmetallic Mineral Products
Lightly polluting industries (8)	Processing of Petroleum, Coking and Processing of Nuclear Fuel, Manufacture of Liquor, Beverages and Refined Tea, Manufacture of Chemical Fibers, Other Manufacture, Manufacture of Textile, Production and Supply of Gas, Mining and Processing of Nonmetal Ores, Manufacture of Foods

**Table 3:** Indices and calculation methods.

Variable	Symbol	Variable Name	Measurement Method
Core explanatory variable	lnERS	Environmental regulation intensity	The “three wastes” (waste gas, wastewater, and solid wastes) are the main pollutants in industrial production. In this study, the intensity of controlling the “three wastes” was used as a proxy variable to reflect the environmental regulation intensity. Due to the serious lack of investment data for solid waste control, the intensity of wastewater control and the intensity of waste gas control were primarily used to determine the environmental regulation intensity. The entropy weight method was used to obtain the regional environmental regulation intensity by calculating the ratio of the total investment in wastewater control to the total amount of industrial wastewater discharge (10,000 yuan/10,000 tons) and the ratio of the total investment in waste gas control to the total volume of industrial waste gas emissions (10,000 yuan/100 million standard cubic meters). Then, the regional environmental regulation intensity was logarithmically processed. The gross domestic product (GDP) deflator was used to eliminate the effect of inflation on the variable.
Controlvariable	lneco	Regional economic development level	The GDP of each province/city/region was selected as a proxy variable for the regional economic development level, and the obtained value was deflated using the consumer price index (CPI) and logarithmically processed.
	lnway	Regional transportation convenience	The highway density in each province/city/region was used as the proxy variable for the degree of transportation convenience and calculated by dividing the total length of highways by the land area. The calculated highway density was logarithmically processed.
	lnedu	Education level	Based on the method described by Wang (2000), the ratio of the regional education level $\left(E_{it}\right)$ to the total population of a region $\left(P_{it}\right)$ was used to measure the education level of a region. $P_{it}$ represents the total population of region i in year t. The total educated population in the region was calculated using the following formula: $E_{it}=6*e_{it_{1}}+9*e_{it_{2}}+12*e_{it_{3}}+16*e_{it_{4}}$$.E_{it}$ represents the number of educated people in region i in year t$.\;e_{it_{1}}$$,\;e_{it_{2}}$$,\;e_{it_{3}}$$,\;\mathrm{and}\;e_{it_{4}}$ represent the number of primary school graduates, the number of middle school graduates, the number of high school graduates, and the number of college graduates or above in region i in year t, respectively. The constants 6, 9, 12, and 16 represent the average years of education received by primary school graduates, middle school graduates, high school graduates, and college graduates or above, respectively.
	lnpop	Market size	The year-end population in each province/city/region was used as a proxy variable for the regional market size and logarithmically processed.
	lnRD	R&D and innovation level	The logarithmic value of the number of patent applications in a region was used to measure the R&D and innovation level in the region.

**Table 4:** Transboundary pollution effects of various types of polluting enterprises and their rates of change.

Nationwide	2000	2013	Rate of Change from 2000 to 2013 (%)
All polluting enterprises	1.2449	1.0859	−12.77
Severely polluting enterprises	2.2302	1.0982	−50.76
Moderately polluting enterprises	1.4135	1.6030	13.41
Lightly polluting enterprises	1.4442	1.4154	−1.99

**Table 5:** Regional differences in transboundary pollution effects.

Type of Pollution	Eastern Region			Central Region			Western Region		
	2000	2013	Growth Rate from 2000 to 2013	2000	2013	Growth Rate from 2000 to 2013	2000	2013	Growth Rate from 2000 to 2013
All pollution	1.0026	0.9319	−7.05%	1.1576	1.1518	−0.50%	1.5253	1.1833	−22.43%
Severe pollution	1.7479	0.995	−43.07%	0.9868	1.0079	2.14%	3.5012	1.2529	−64.21%
Moderate pollution	0.9661	1.8297	89.40%	1.4418	1.3077	−9.30%	1.8048	1.5921	−11.78%
Light pollution	1.6299	1.5501	−4.90%	1.5412	1.2902	−16.29%	1.2094	1.3755	13.74%

**Table 6:** Empirical results.

	Fixed Effects (1)	Random Effects (2)
lnERS	−0.0468	−0.0645 *
lneco	−0.329 **	−0.137 *
lnway	0.116 *	0.116 **
lnedu	−0.0316	−0.0044
lnpop	−0.1010	0.0119
lnRD	−0.0100	−0.0969 *
Constant	4.243 **	2.113 **
*p* value (F value)	0.0068	0.0045
Hausman test	3.26	
Hausman (*p* value)	0.1963	
Observations	90	
Number of years	3	

Note: Standard errors in parentheses; * *p* < 0.1, ** *p* < 0.05, *** *p* < 0.01.
